# The effect of volunteering and voluntary group membership on student's persistence

**DOI:** 10.1016/j.heliyon.2021.e07900

**Published:** 2021-08-30

**Authors:** Gabriella Pusztai, Hajnalka Fényes, Valéria Markos

**Affiliations:** a University of Debrecen, Center for Higher Education Research and Development, Debrecen, Hungary; b University of Debrecen, Department of Sociology and Social Policy, Debrecen, Hungary

**Keywords:** Volunteering, Voluntary organization and group membership, Persistence, Higher education students, Quantitative analysis

## Abstract

In the literature on higher education, civic involvement is discussed in connection with civic education or as a protective factor against attrition. In a series of surveys, we have followed higher education students' volunteering at several higher education institutions of Central and Eastern Europe since 2005. Based on Wilson (2000), we distinguish between formal volunteering and volunteer work which does not require organizational membership. Among young people, volunteer work is rather a program-centered, personally motivated, and individual activity (Inglehart 2003). In this study, however, we also examine students' voluntary group membership, which could provide a potential framework for volunteer work. Through a stepwise linear regression model, we analyze the effect of volunteering and voluntary group membership on students' higher education persistence. The analysis is carried out on data from the PERSIST 2019 survey (N = 2199). We find that persistent academic progress is more common among women, individually well-off students from objectively less affluent families, those who pray regularly, those who have a close relationship with their parents, professors, and peers, and those have done volunteer work during their higher education studies.

## 1 Introduction

In the analysis of persistent academic progress in higher education, it is increasingly common to investigate the institutional impact of higher education, that is, the way in which the institutional curriculum, co-curriculum, and extracurriculum contribute to elevating the likelihood of successful graduation. The separate role of institutional contribution was first formulated by Astin's IEO (Input-Environment-Output) model. The extensive literature of valuable empirical findings based on Tinto's student integration model also underscores the importance of uncovering institutional tools, possibilities, and policies. In this study, we analyze how students' voluntary work and their membership in groups and organizations contribute to their persistent academic progress.

We begin the analysis by discussing the definition of and research findings about persistence, followed by an overview of studies on students' volunteer work and voluntary organization and group membership, complemented with a summary of theories and findings with respect to how volunteering and voluntary group membership could provide support in one's studies. In addition, we also present the general characteristics, trends, and influencing factors of civic engagement. Subsequently, we formulate our hypothesis about the factors behind persistence based on the literature, which we test through stepwise linear regression analysis.

## 2 Student persistence

Higher education retention and student persistence are two sides of the same coin, so they cannot be understood separately. Retention reflects the institutional perspective and measures the proportion of those in a student cohort who continue their studies in the following year; in other words, those who register for the respective semesters year after year (Mortenson 2012). This is a statistical indicator, which characterizes a higher education system, institution, or program and might also be interpreted as an indicator of quality (Seidman 2012). In contrast, student persistence considers progress from the students' perspective and shows individual determination to go on and to achieve goals and graduate successfully. This comprises the action of deciding as well as the activities which lead the student closer to graduation (Berger and Milem 1999; Seidman 2012). What is described here is not student persistence but persistent progress, which is the opposite of what happens when students do not register for the following semester (attrition). This may occur voluntarily, whereby students discontinue their studies temporarily (stopout) or leave the institution (withdrawal), or involuntarily, if the institution does not allow the continuation of their studies (dismissal). Invariably, the risk of failing to graduate is present (dropout). Even if students do continue their studies at the same institution or at another at a later date, it cannot be regarded persistent progress.

Retention was first examined in the 1960s, and it was soon revealed that retention rates varied by institution. Consequently, research began into the effect of the institution on students (Feldmann and Newcomb, 1969; Spady 1970), with two main explanations emerging. Astin, who constructed the model of student involvement, highlighted (1984) the importance of students' actions as opposed to their beliefs or thoughts. Tinto's (1993) interactionist theory argues that students, who are in a permanent position of deliberation, make the decision to stay in or drop out based on their everyday experiences and interactions. Tinto's concept focuses on the students' perceptions and interpretation of their own situation, which also affects how students interpret each other's activities. Milem and Berger (1997) remark that actual behavior is of secondary significance in Tinto's concept. Various subsequent studies feature the views influenced by students' higher education contacts (Pascarella and Terenzini 2005; Pusztai 2015). French (2017) improved Tinto's model on persistence and combined it with social change model (SCM) of leadership development. The positive outcome of successful implementation of SCM is that it encourages students to work for the common good. All in all the latest empirical findings (Marley and Wilcox 2021) supported the relevance of Tinto's concept and found that social integration has a positive effect on success and persistence of students.

## 3 Factors affecting persistence

Numerous studies have sought to explain the differences which are observed in persistence (Pascarella and Terezini, 2005). Some explanations of persistence point to students' demographic and socio-economic characteristics and past academic performance, while others emphasize the attributes of the higher education institution. In addition, the different demographic, social, and institutional variables may interact with each other (Peltier et al., 2000).

As for individual characteristics, persistence varies as a function of gender and age (Aina 2013; McNabb et al. 2002; Wolter et al., 2014; Pusztai 2019). In short, studies from the 20^th^ century mostly point to females' lower persistence, while evidence on females' advantage has been increasingly common since the 2000s (Hu & St. John 2001). There are various explanations as to why low-status and first-generation students are less persistent (Stinebrickner and Stinebrickner, 2014; Hernández et al., 2017). Many have found a positive link between the family's financial situation and persistence (Alon 2011; Chen 2012). It is not clear, however, whether persistent progress is helped or hindered by student employment (Riggert et al., 2006; Perna 2012; Kocsis and Pusztai 2019). Some studies even suggest that student loans might have a negative effect on low-status students' persistence (Herzog 2018). Certain student groups might achieve their academic goals better if they have a close relationship with their parents (Wartman and Savage 2015).

Religiosity may support academic performance through multiple channels. Private religious practices (e.g., prayer) could assist work discipline and perseverance towards study goals through the self-control over individual activities, and could help students in concentrating on their studies and resisting diversions (Saroglou 2011; Carol and Schulz 2018). Sherkat (2003) argues that membership in religious community might even increase attrition risk by reducing social and academic integration within the higher education institution. In contrast, social capital accumulated by religious groups has a positive influence on higher education studies and effectively compensates disadvantages of low or minority status (Coleman 1988; Pusztai 2015), although in some cases religious group membership neither improves nor deteriorates the likelihood of degree completion (Baker 2008).

Another series of explanations consider institutional characteristics. Early research on attrition focused on the attachment to the academic and other social communities at the institution, with a link found between the perseverance with one's studies and the attachment to the institution (Tinto 1993). If students fail to leave communities which were decisive to them before the entry to higher education, they cannot be integrated into the higher education institution (Astin 1984; Kuh 2009; Pascarella and Terenzini 2005). Researchers also highlight the relationship with professors as well as the time spent on campus as a decisive indicator of students' higher education integration (Bean and Breadley, 1986; Astin 1993). Some write about “involving colleges”, which contribute to the attachment to the institution and degree completion and assist persistent progress (Kuh et al. 1991). An effective way of students' social integration is the participation in voluntary activities together with peers, which elevates the likelihood of persistent progress. Milem and Berger (1997) are among the many authors who consider volunteer and civic involvement to be part of peer involvement.

The resources derived from relationships to groups, organizations, and actors at the institution are often categorized as social capital (Kim and Schneider 2005; Perna and Titus 2005; Altbach 2009; Pusztai 2015). Findings from the Central and Eastern European region show that, of all voluntary activities within the institution, intergenerational ones increase persistence significantly, while intragenerational ones often exert a negative effect. In contrast, membership in outside voluntary groups mostly has a positive impact, regardless of whether they are intragenerational or intergenerational (Pusztai 2015).

While Astin's main focus is on the time spent on campus, Baker (2008) remarks that it is not the time spent with an activity which influences performance significantly but rather the type of the activity. Baker's classification consists of six fields of student activities within the framework of voluntary organization and group membership, which contribute to performance to varying degrees. In general, involvement in political, religious, and artistic organizations has a positive effect on academic performance (Astin et al. 2011; Pusztai 2015). However, the effect of sports and student organizations is ambiguous (Kovács 2019). Findings in a Central and Eastern European region, reveal that membership in a voluntary organization and involvement in a religious community outside the institution strengthen persistence. It is found that civic engagement (the complex joint variable of organizational involvement and volunteering) neither increases nor decreases persistence once all factors are considered (Fényes et al., 2018).

## 4 Individual and collective forms of civic engagement

The most important attribute of volunteering is that it is not a compulsory activity; it is aimed at the benefit of others (individuals, organizations, or the entire society); no remuneration is offered for it; and it mostly occurs in an organized framework. Voicu and Voicu (2003) provide a narrower definition by restricting volunteering to volunteer work within a formal (organizational) framework. Wilson (2000) separates program volunteering, which does not require membership in an organization, from associational volunteering, which is what members of an organization take part in. Inglehart's (2003) findings reveal that young people today prefer to do volunteer work in new, more flexible, and less constant organizational frameworks (mostly in charity and sports organizations). The category of leisure volunteering is also used by some researchers to reflect the decreasing altruistic nature and increasing subjective value of volunteering, including the need for and motivation towards joy and entertainment (Stebbins 1996). Activities of leisure volunteering serve the good and interests of others and those of the volunteer simultaneously (Kaplan 1975). Naturally, a leisure activity only constitutes volunteer work if it includes an action in the given organization for the benefit of others.

Wilson (2000) highlights that greater financial capital, higher level of education, more extensive social network, greater organizational involvement, and religious activity (mostly collective religious practices, see Hodgkinson 2003) all increase the likelihood of volunteering in the adult population. In addition, Perpék (2012) underscores that social resources have a larger impact on volunteering than socio-demographic variables. The likelihood of volunteering is elevated by a more extensive circle of friends, more formal and informal interactions, stronger family relationships, religiosity as an indicator of social capital, membership in organizations, broad personal social network, and trust in others (Perpék 2012). As for the strength of relationships, it can be observed that the willingness to do volunteer work is increased mainly by weak ties (acquaintances, workplace relations, relationships across multiple social groups) and by trust among people (Voicu and Voicu 2003).

There are diverging trends in young people's civic participation across countries (Flash Eurobarometer 2019, N = 10,786). Of all young people aged 15–30, over a half have taken part in volunteering activities in Ireland, Denmark, and the United Kingdom. Croatia, Sweden, and Malta have the lowest share of volunteers in the EU. In Hungary, 44% of respondents have done volunteer work, which is below the EU average (58%). As for youth organization membership, the proportion of members is the highest in Ireland, Sweden, Finland, Luxemburg, and Belgium and the lowest in Croatia, Hungary, and Romania. Thus, the divergence between Western Europe and Central and Eastern Europe is also apparent with respect to civic engagement. In Western countries, where democracy has a long-standing tradition and civil society is also more developed, the prevalence of volunteering and civic organization membership is greater than in Eastern countries (Juknevičiusa and Savicka, 2003; Flash Eurobarometer 2019).

Higher education students are to become the graduates and leaders of the future, so it is vital to follow the trends among them. According to our series of surveys encompassing five countries from Central and Eastern Europe, the prevalence of volunteering has been gradually on the rise since 2005. At the University of Debrecen (Hungary), the proportion of regular volunteers more than doubled between 2005 and 2010 (3–6%), with 26% of students in 2010 having done volunteer work at some point during their studies. By 2015, when the sample also included areas outside the Hungarian border, 38.82% of students had done volunteer work during their studies. By 2019 (see the present study), the figure had grown to 45.3% (Fényes & Pusztai 2012a, 2012b; Bocsi and Fényes 2012; Fényes 2015; Markos 2018).

The prevalence of volunteering among higher education students is influenced by various factors. Findings of a regression analysis using 2010 data from the University of Debrecen (Hungary) reveal that the likelihood of volunteering is increased by the mother's higher educational attainment, the student's better individual financial situation, and religiosity (when considering active church members and those who are religious in their own way together) (Fényes and Pusztai 2012b). Data from 2014, gathered in the same region of Central and Easter Europe as the data for this study, show that volunteering is more common among women, people facing financial difficulties, urban residents, and those who have a rather casual relationship with their parents but a closer one with their professors (Fényes 2015).

As demonstrated, membership in associations is relatively low in Central and Eastern Europe compared to Western Europe, in a similar way to volunteering. In the Hungarian adult population, volunteer work is mostly done in religious, youth, leisure, recreational (sports), healthcare, educational, professional, and cultural organizations. Importantly, the number of non-profit organizations which enable volunteering is still low (Bartal 2010).

According to our previous research findings, 12.2% of students at the University of Debrecen were involved in religious organizations in 2010. The most popular student organizations were sports clubs and associations (12.2%), cultural groups (9.8%), and student representative groups (6%). Furthermore, 5.2% of students took part in voluntary organizations, 3.4% in non-governmental organizations, and 1.9% in political organizations or parties (Fényes and Pusztai 2012b). These proportions seem to be on the rise (see the empirical section of this study).

Voluntary group membership is more frequent among higher education students who have minority status, whose father has a higher education degree, who consume high culture in the Bourdieuan sense regularly, and who maintain a close social network with professors. Group membership does not seem to correlate with financial situation and the place of residence. The study also points out that active involvement in organizations has a larger effect on students' performance than socio-economic variables have (Pusztai 2015). The joint likelihood of students' voluntary group membership and volunteering (measured by a civic engagement index) is increased by a favorable financial situation, rural place of residence, good relationships with faculty and peers, and religiosity (Fényes et al., 2018, Pusztai et al., 2019).

## 5 Hypotheses

In our empirical analysis, we employ linear regression models to uncover effects on persistence. Based on the literature, we formulate the following hypotheses.

H1: According to Astin's (1984) involvement theory and Tinto's (1993) theory on integration, involvement in organizations outside the institution could weaken on-campus ties and could thereby decrease persistence. In contrast, Inglehart (2003) argues that volunteering is rather an individual activity for young people, without membership in an organization, which is why the pull-effect of membership in external organizations does not exists. Moreover, Milem and Berger (1997) suggest that volunteering strengthens higher education students' persistence, whether it is organized or not. Based on this, we hypothesize that volunteering increases persistence.

H2: In this study, we regard students' participation in organizations and groups as a potential framework for volunteering. Based on results from Astin (1984), Tinto (1993), and Milem and Berger (1997), we hypothesize that the composite index created from students' group membership, comprising organizations both within and outside the higher education institution, neither increases nor decreases persistence.

H3: Based on Isphording and Qendrai (2019) we hypothesize that females are more persistent than males, possibly because females want to correspond to traditional gender roles and external expectations (from professors and parents) to a greater degree than males.

H4: Concerning social background, we hypothesize that well-educated parents' children are more persistent than their peers (Stinebrickner and Stinebrickner, 2014; Hernández et al., 2017), but favorable financial background rather decreases persistence because status striving is milder in their case, and greater educational experimentation, resulting in poorer persistence (Pusztai 2019). We presume as well, that the student's individual financial situation also effects persistence, and student employment and student loans might have a negative effect on persistence (Riggert et al., 2006; Herzog 2018).

H5A: We hypothesize that persistence is enhanced by both private and collective forms of religiosity (Astin et al., 2011; Saroglou 2011; Carol and Schulz 2018).

H5B: According to our alternative hypothesis, collective religious practices may even decrease persistence because it distracts from the social and academic integration within the institution (Sherkat 2003).

H6: We hypothesize that students' social resources, for example intergenerational relationships with faculty at institution (Bean and Breadley, 1986; Astin 1993); a close relationship with the parents (Wartman and Savage 2015; Pusztai 2019); and a strong relationship with peers (Berger and Milem 1999).strengthen persistence. However, close relationship with friends who are not part of the institution weakens institutional social integration and thereby decreases persistence (Tinto 1993; Astin 1993; Kuh 2009; Pascarella and Terenzini 2005).

## 6 Data, variables, and methods

The database consists of a large-sample student survey^1^ (N = 2199), conducted in the academic year 2018/19. The survey was carried out at higher education institutions in Eastern Hungary^2^ and in four other countries^3^ (Slovakia, Romania, Ukraine, Serbia). The Hungarian subsample (N = 1034) was collected using quota sampling and is representative with respect to faculty, field of study, and form of funding. At institutions outside Hungary, the aim was probability sampling: groups of students in university/college courses were selected and surveyed exhaustively (N = 1165). The sample consists of full-time bachelor's students in their second year and of second-year or third-year students from undivided programs which offer a master's degree.

This analysis employs the method of linear regression to uncover the factors which influence students' persistence. Our central research questions asks whether persistence is increased or decreased by students' volunteer work and voluntary organization and group membership when additional social background variables are taken into account. Since there is potential correlation between volunteering, group memberships, and social background variables resulting in multicollinearity, stepwise inclusion is used. Besides students' voluntary organization and group membership and volunteer work, explanatory variables also include gender, students' social background, religiosity, and students' social resources.

Our research group has measured persistence using a four-item series of questions since 2015, based on French et al. (2005). The dependent variable is the principal component of persistence, with 56.7% of the total variance explained and a Cronbach's alpha value of 0.74. The scale is constructed from the following four items: my studies will be useful in my professional career; I am very committed to graduating; I aim for the best academic results possible; I do everything in my power to attend lectures, seminars, and practice sessions. A 1–4 Likert scale represents agreement or disagreement with the items.

The explanatory variable indicating students' volunteering is measured by the fact whether they have done volunteer work during their higher education studies (1: has, 0: has not; 45.3% of respondents have). Voluntary organization and group membership is quantified through an index (0–8), compiled from different organization and group memberships.^4^ Respondents in the sample are involved in 0.88 groups or organizations on average (standard deviation is 1.26), which is relatively low and reveals the underdevelopment of civic engagement in the region. The next explanatory variable is gender, which has the following distribution in the sample: 30.1% male and 69.9% female (1: male, 0: female). We proxy social background using the father's years of education (with a mean of 12.7 and a standard deviation of 2.54) and mother's years of education (with a mean of 12.9 and a standard deviation of 2.6) and four indicators of financial status. The family's financial situation was measured by the possession of durable consumer goods^5^ (objective financial situation index 0–9, with a mean of 6.9 and a standard deviation of 1.63) and by a relative financial situation indicator, which compares the family's financial situation to the student's peers (on a 1–5 scale where 3 is the average situation, with a mean of 3.3 and a standard deviation of 0.77). To capture the students' individual financial situation objectively, we created a composite index indicating the possession of durable goods^6^ (0–6, with a mean of 1.8 and a standard deviation of 1.5) and a subjective indicator of individual financial situation^7^ exploring whether the student can afford a significant purchase or is unable to cover even the basic expenses (1–4, with a mean of 3.2 and a standard deviation of 0.62). Finally, the variable for the place of residence at the age of 14 (1: urban, 0: rural) is also included (with 62.2% of respondents as urban residents).

Religiosity is represented by two indicators. First, individual religiosity is measured by the frequency of private prayer (1–7, with a mean of 3.8 and a standard deviation of 2.29); second, collective religiosity is measured by the frequency of church visits (1–5, with a mean of 2.5 and a standard deviation of 1.28). Students’ social resources are represented by four indices, measuring the frequency of social activities with parents^8^ (6–30, with a mean of 19.6 and a standard deviation of 3.44), faculty^9^ (0–18, with a mean of 4.2 and a standard deviation of 4.19), fellow students from the same program or institution^10^ (0–11, with a mean of 8.3 and a standard deviation of 2.84), and friends outside the institution^11^ (0–11, with a mean of 7.7 and a standard deviation of 3.16).

## 7 Results of the survey

According to previous findings from a survey carried out in 2010 at the University of Debrecen (Hungary) on a less extensive sample (see Fényes and Pusztai 2012b), 12.2% of students were involved in a religious organization, which is 24% in the current sample. There has been a slight increase in sports association or club membership (from 12.2% to 13.2%). Cultural (art) group membership has declined slightly (from 9.8% to 8%), while youth representative membership has risen somewhat (from 6% to 11.1%). There has also been an increase in the participation in political organizations (from 1.9% to 3.2%), but the fraction of politically active students is still low. In contrast, students' involvement in charitable and non-governmental organizations has increased substantially, from 3.4% to 13.2% and from 5.2% to 10.8%, respectively.

We examined the correlation between volunteering and voluntary group membership in the sample. The results reveal that 45.3% of students have done volunteer work during higher education, yet the proportion of voluntary group members is significantly lower (see above), with students participating in 0.88 groups on average according to the group membership index. As the two-sample t-test suggests, those who have done volunteer work are involved in 1.34 organizations on average, while the same figure is only 0.49 for those who have not done volunteer work (p = 0.000), which implies a correlation between volunteering and voluntary group membership.

The following analysis uncovers the factors which influence students' persistence using linear regression. To address potential multicollinearity between explanatory variables, stepwise inclusion was employed. The model first included the index for voluntary organization and group membership, followed by students' volunteering, gender and social background variables, two variables of religiosity, and finally completed by variables of social capital. In the regression models, the explanatory power is relatively low. However, we did not attempt to create a comprehensive model by including all explanatory variables; instead, our intention has been to investigate the direction and significance of effects exerted by certain featured variables, with special regard to the effects of students' voluntary group membership and volunteering on persistence (Table 1).

**Table 1:** Effects on the principal component of persistence (linear regression coefficients and their significance, by stepwise inclusion of explanatory variables; ∗ marks significance between 0.01 and 0.05, ∗∗ marks significance between 0.001 and 0.01, and ∗∗∗ marks significance below 0.001) (N = 2199).

	Beta	Beta	Beta	Beta	Beta
Voluntary group membership index	0.03	0.004	-0.002	-0.034	-0.035
Volunteering		0.076∗∗	0.081∗∗	0.073∗∗	0.061∗
Gender (1: male)			-0.150∗∗∗	-0.130∗∗∗	-0.115∗∗∗
Mother's years of education			-0.004	-0.004	-0.012
Father's years of education			0.014	0.013	0.012
Objective financial situation index (family)			-0.053	-0.028	-0.058∗
Relative financial situation of the family			0.023	0.011	-0.010
Objective financial situation index (student)			0.065∗	0.053	0.059∗
Subjective financial situation of the student			0.068∗	0.069∗	0.055∗
Place of residence at the age of 14 (1: urban)			-0.013	0.008	0.013
Private religiosity				0.145∗∗∗	0.122∗∗
Collective religiosity				-0.013	-0.027
Index for the relationship with parents					0.180∗∗∗
Index for the relationship with faculty					0.067∗
Index for the relationship with university peers					0.076∗∗
Index for the relationship with external friends					0.004
Adjusted R-squared	0.000	0.005	0.037	0.053	0.1

The results suggest that the index of voluntary organization and group membership does not affect persistence (the effect is not significant in any of the regression models). Consequently, active group membership does not act as a pull-factor from the campus and, therefore, does not decrease persistence. In contrast, volunteering increases students' persistence in the examined region unambiguously; its effect hardly changes upon the inclusion of additional explanatory variables. Furthermore, women, individually well-off students from objectively less affluent families, those who pray regularly, and those who have a close relationship with their parents, lecturers, and peers all display greater commitment towards the completion of their studies. In sum, volunteering indeed provides protection against attrition, while group membership has no effect, that is, does not decrease persistence. Interestingly, persistence is not weakened by a close relationship with external friends and by regular church visits (as sources of external relationships), so they do not constitute a pull-factor. The parents’ educational attainment has no separate impact on persistence once the effect of financial situation is taken into account.

## 8 Discussion

As established, the proportion of students who do volunteer work has risen significantly in the examined region (with 45.3% of students doing volunteer work during their higher education studies), although it has not reached the levels customary in Western Europe. The increase in volunteering might be the consequence of the introduction of school community service at Hungarian secondary schools in 2012, which compels each secondary student to engage in 50 h of community service until graduation. Preliminary studies show the positive effect of school community service on subsequent volunteering (Markos 2020). Another reason for the increasing prevalence of volunteering in the examined region as well as in Western Europe could be the rise of the novel, career-focused volunteering among young people. Although this study does not investigate the motivations behind volunteering, we presume that, besides traditional volunteering, which has the objective of helping others, the appearance of career-focused volunteers has also contributed to the rise of volunteering among young people in the region. In addition, we show in previous studies which explore student clusters by motives of volunteering that there is a group of students who derive no motivation to pursue volunteer work from their friends' and family members’ voluntary activity. Thus, the growing popularity of volunteering among friends and family members might provide another reason for the increase in voluntary activity. Finally, the inclusion of voluntary experience in the curriculum vitae, which in 2015 was hardly an influential motivation for students to pursue volunteer work, might be of greater importance nowadays, when employers in the region take into account volunteer experience more readily in the examined region (Bocsi et al., 2017).

Students' participation in organizations is still very low, however. On average, students are involved in 0.88 organizations, which might be explained by the relatively short history of democracy in the post-socialist countries of the region, by underdeveloped non-profit sector, and by the scarcity and relative unpopularity of groups and organizations at higher education institutions. Nonetheless, we can observe an upward trend. Compared to 2010, students' participation in religious groups, which is the most popular group type, has more than doubled (to 24%), while involvement in charitable and other non-governmental organizations has also doubled (to 10–12%). Overall, this study considers students' group membership as a potential framework for volunteering. Since group membership and volunteering are correlated, we included these two variables in the regression models of persistence in two steps to address multicollinearity.

Our regression models first examined the main research question of this study, namely the effect of volunteering and group membership on persistence. In accordance with our first hypothesis (H1), volunteering increases persistence both before and after the inclusion of other background variables. This is consistent with the view that volunteering among young people nowadays rather constitutes a program-centered individual activity (Inglehart 2003), whereby the pull-effect of membership in external organizations does not materialize as suggested in the models by Astin (1984) and Tinto (1993). Similarly, it is in accordance with our second hypothesis (H2) that persistence is neither positively nor negatively affected by students' membership in organizations within and outside the institution, which we explain by the mutual neutralization of the simultaneous pull and push factors of different group memberships. This finding is somewhat contrary to the suggestion put forward by Milem and Berger (1997), namely that civic involvement clearly increases persistence even if it is carried out within an organizational scheme. We argue that the difference is due to our consideration of group membership as a potential framework for volunteering and the lack of a variable which would unambiguously separate formal and informal volunteering and would make the distinction between volunteer work in organizations and mere group memberships.

Our findings also show females' greater persistence as opposed to males, which is in accordance with our third hypothesis (H3). Concerning social background (H4) our findings suggest that the effect of the parents' educational attainment on persistence is neither positive nor negative, but family's objective financial situation has a negative impact on persistence, which implies that children of less affluent parents are more persistent. We found as well, that students' better individual financial situation does in fact increase persistence. Concerning the effect of religiosity on persistence (H5), we show the positive effect of regular prayer on persistence. However, regular churchgoing seems to leave persistence unaffected, which is slightly at odds with findings revealing a positive effect, by Astin et al. (2011). Our alternative hypothesis, which states that collective religiosity might decrease persistence as it could weaken social integration on campus (Sherkat 2003), cannot be corroborated, as well. These findings are parallel to the results regarding the first two hypotheses, namely that volunteering, which mostly an individual activity is; increases persistence but group membership (a collective activity) exerts no effect. In accordance with our final hypothesis (H6), students' persistence is strengthened by close relationships with faculty, parents and fellow students. In contrast, external friendships do not act as a pull-factor from campus and neither decrease nor increase persistence, which is contrary to our hypothesis.

The limitation of the study that we were not able to examine the effect of formal and informal voluntary activity of students' on persistence separately, instead we have two variables in the database: volunteering and voluntary group membership of students. We supposed that voluntary group membership is a potential place for volunteering but we do not have exact data on formal volunteering of students. Further limitation of our study that our questionnaire did not differentiate between on-campus and off-campus voluntary group membership, which can have different effect on persistence based on the literature. The third limitation is that out method is quantitative. Qualitative interviews could give more detailed and deep information about our research question. We have gathered qualitative interviews focusing on the risk of attrition of students', but volunteering was not mentioned neither a risk factor, nor a supporting factor of persistence. In frame of qualitative data collection focusing directly on our research question we could reveal mediating mechanism of the support provided by volunteering on preventing attrition.

## 9 Policy recommendations

Our findings reveal that students who have done volunteer work during their higher education studies are more persistent. Furthermore, volunteering among higher education students has become more frequent since the introduction of school community service in Hungary. It would also be necessary to guide higher education students towards volunteering to increase their persistence, among other goals. The first step to achieving this could be the inclusion of service learning courses in the curriculum, perhaps even in a compulsory manner, as it has been successfully carried out in public education. Besides curricular service learning, the expansion of extracurricular opportunities would also be necessary through, say, operating career offices at higher education institutions, where trained professionals may offer career-focused volunteer work opportunities to students. It is also revealed that students' participation in organizations is still very low in the investigated region, even though group memberships within and outside the institution can be considered as a potential framework for volunteering. For instance, religious youth organizations provide the possibility of charitable and volunteer work as well as a sort of civic education, which should be assisted by universities through, say, the allocation of appropriate venues. It would not be needed for higher education institutions to contribute financially to religious youth communities because churches support them by providing trained personnel and funding for programs. In the examined region, most sports clubs focus their resources on professional teams, which do not include higher education students. It would be important for clubs to support the interests of amateur students' players, who take part mainly in active leisure sports activities, so as to enable the contribution of sports club memberships to the integration to campus life. It would be vital to popularize the establishment of cultural (art) groups, which can improve social skills, enhance creativity, and compensate disadvantages in cultural capital. This could occur either in a co-curricular manner or as an elective course for academic credits and would provide a potential framework for volunteer work. To strengthen persistence, it would be worthwhile to involve parents and faculty in the activities of the groups mentioned above, since it is apparent that persistence is increased by both intragenerational and intergenerational relationships.

## Declarations

### Author contribution statement

Gabriella Pusztai: Conceived and designed the experiments; Performed the experiments; Contributed reagents, materials, analysis tools or data; Wrote the paper.

Hajnalka Fényes, Valéria Markos: Performed the experiments; Analyzed and interpreted the data; Wrote the paper.

### Funding statement

This work was supported by the 10.13039/501100012550National Research, Development and Innovation Fund of Hungary (K_17 funding scheme).

### Data availability statement

Data included in article.

### Declaration of interests statement

The authors declare no conflict of interest.

### Additional information

No additional information is available for this paper.

## Footnotes

1 The title of the research project was “The Role of Social and Organizational Factors in Student Attrition”. The authors are the members of the Center for Higher Education Research and Development (CHERD-Hungary), which organized the research project and managed the data collection.

2 University of Debrecen, University of Nyíregyháza, Debrecen Reformed Theological University, Saint Athanasius Greek Catholic Theological College.

3 Babeş-Bolyai University (BBTE), Emanuel University of Oradea, Ferenc Rákóczi II Transcarpathian Hungarian College of Higher Education, Constantine the Philosopher University in Nitra, Mukachevo State University, University of Oradea, Partium Christian University (PKE), Sapientia Hungarian University of Transylvania, J. Selye University, University of Novi Sad, Uzhhorod National University.

4 Non-governmental organization, sports club or association, religious organization or small religious community, political organization (party, movement), student union or other representative membership, art group, charitable organization, other group or organization.

5 Components of the index: Does the family possess an apartment or house, a five-year-old car or younger, a flat-screen television, a personal computer or laptop with broadband internet access at home, a tablet or e-book reader, mobile internet (on the phone or computer), a dishwasher, an air-conditioner, and a smartphone?.

6 Components of the index: Does the student possess an apartment or house, a car, an above-average smartphone (e.g. iPhone), an above-average computer or laptop, a tablet or e-book reader, and savings for house purchase?.

7 1: Often I do not have enough money for basic everyday necessities. 2: Sometimes I do not have enough money for everyday expenditures. 3: I have everything I need but cannot afford larger expenditures. 4: I have everything I need and can also afford larger expenditures.

8 During the years spent in higher education, have your parents done any of the following activities with you? Having a conversation; asking about the studies and exams; providing financial support; planning activities together; planning sports activities together (the frequency of the activities is specified on a 1–5 scale, with Cronbach's Alpha at 0.793).

9 Do you have a professor or lecturer with whom you do any of the following activities? Talking about the curriculum outside lectures; talking about topics not specified in the curriculum; talking about literature or art; talking about questions of politics; talking about private matters; talking about plans for the future; maintaining regular e-mail conversations; paying special attention to the career; talking about sport and healthy lifestyle (1: there is one such professor, 2: there are more, 0: there is none; with Cronbach's Alpha at 0.879).

10 Do you have a fellow student in the program or at the institution with whom you do the following activities? Talking about academic problems; talking about private matters; spending leisure time together frequently; discussing future plans; visiting in case of illness; borrowing textbooks or study material; talking about scientific questions; talking about culture or questions of politics; talking about art; studying together; doing sports together (1: there is, 0: there is not; with Cronbach's Alpha at 0.842).

11 Do you have a friend outside the institution with whom you do the following activities? See the list for fellow students (1: there is, 0: there is not; with Cronbach's Alpha at 0.873).
